# Supplementary material for: Caenorhabditis elegans models for striated muscle disorders caused by missense variants of human LMNA
Source: PLoS Genet. 2023 Aug 25;19(8):e1010895. doi: 10.1371/journal.pgen.1010895 (PMC10484454; doi:10.1371/journal.pgen.1010895)
Supplement: Attachment — Submitted filename: Response to reviewers.docx [file pgen.1010895.s007.docx]

Dear Orna,

Thank you for a thorough and prompt review by two reviewers and yourself. We are happy that the manuscript was appreciated by all and look forward to hopefully publishing a revised version in *PLOS Genetics.* The suggestions were very helpful and significantly improved the manuscript. Here, we submit a revised manuscript for your consideration. The changes to the text in the revised manuscript are in blue font. Below is our detailed response to the editor’s and reviewers’ comments. The original editor and reviewer comments are in black font. Our responses are in blue font.

We look forward to hearing from you.

Sincerely,

Dan Starr (on behalf of all the authors)

**Editor’s comments:**

The manuscript was fully evaluated at the editorial level and by two independent peer reviewers. The reviewers appreciated the attention to an important topic but identified some concerns that we ask you address in a revised manuscript.

As you will see, the reviewers appreciated the importance of the topic and found your study to be well designed, technically sound and of interest to the field. They did, however, raise several concerns that should be addressed prior to further consideration, and I also added a couple of suggestions/questions. In particular, please note the following:

We thank the editor and the reviewers for carefully thinking about our manuscript and making it better. We have addressed your comments as detailed below.

1. The reviewers appreciated the use of CRISPR/Cas to generate mutant alleles expressed at physiological levels. However, given the possibility of off-target mutations, Reviewer #2 wondered whether and how many times these strains were out-crossed. Alternatively, if you used several independently isolated mutant strains and they all had the same phenotype, that would also address this concern.

We did the following to minimize off-target effects: (1) All guide RNAs were chosen using established algorithms designed to reduce off-target effects (both CRISPR optimal target finder (Gratz et al., 2014 *Genetics*) and CRISPRscan (Moreno-Mateos et al., 2015 *Nature Methods*)). A sentence was added to lines 391-392 in the Materials and Methods to indicate this. (2) The CRISPR/Cas9 mutant lines were backcrossed one time for many of our assays when we introduced GFP markers, the strains are listed in Table 1. (3) Many of the phenotypes were roughly similar for multiple point mutations.

We are less worried about CRISPR/Cas9 off target effects in *C. elegans* as compared to EMS mutagenesis. A paper from Don Moreman’s lab (Au et al., 2019 *G3*) used whole-genome sequencing to address the levels of off-target effects. They sequenced 8 lines where they made CRISPR-Cas9 indel mutations and found a total of 7 single nucleotide variants and one off-target indel. They concluded “We did not detect any off-site variants above the natural spontaneous mutation rate and therefore conclude that this modified protocol does not generate off-target events to any significant degree in C. elegans.” Similarly, Paix et al., 2014 in *Genetics* did not find any off-target mutations above background in whole-genome sequence of 5 strains. Both studies found mutation rates at background levels. We argue that backcrossing many CRISPR/Cas9 generated strains may not be worth the labor. We have not added this discussion to the manuscript, as it is tangential to our main findings.

2. Reviewer #2 noted that the wild type controls used for comparison with the homozygous mutants were themselves not derived from balanced strains, and reviewer #1 also wondered about a possible contribution form the balancer. In this regard, future readers of this study will likely include individuals who are not familiar with the concept of balanced strains. It might be helpful to explain how this works, and specifically the effect of maternally contributed wild type protein on to the ability of the mutant strains to reach adulthood.

We thank the reviewer, as addressing the potential contribution of the balancer in our experiments is an important control. We looked at the balancer over wildtype (referred to as hT2/+ in the paper) in our swimming (Fig. 3), nuclear migration (Fig. 4), and nuclear morphology (Fig. 6) assays. In all three cases, the hT2/+ control was not statistically different from wild type controls. We now include these data in the revised Figs. 3, 4, and 6. We refer to the hT2 balancer in the three revised figure legends.

3. Both reviewers noted that the study does not adequately address the issue of micronuclei, and Reviewer #1 also wondered whether the nucleoplasmic marker you used is sufficient for detecting micronuclei, blebbing and chromosome bridges.

We acknowledge that our analysis of nuclear morphology was cursory. However, a more detailed analyses are beyond the scope of this manuscript. The marker we used is a soluble nuclear nls::GFP::lacZ fusion protein with the SV40 NLS (originally from the 1995 Fire vector kit) and used by many in the field. We added this information to the Figure 6A legend.

We agree with the reviewers that a soluble nucleoplasmic marker might not be sufficient to see all micronuclei or chromatin bridges. Throughout the manuscript, but especially in the paragraph from lines 284-293, we changed the various terms to be less specific. We now refer more non-specifically to “nuclear morphology defects” or “blebs.”

4. As *C. elegans* has only a single lamin gene, a more detailed discussion on its similarities to Lamin A/C vs. Lamin B is warranted, as noted by Reviewer #1. This is especially relevant because you are modeling Lamin A/C mutations in LMN-1, which has features that resemble Lamin B.

It remains difficult to classify *C. elegans* LMN-1 as either an A or B-type lamin. A- and B-type lamins separated from one another in the vertebrate lineage. All invertebrate lamins are equally related to both A- and B-type lamins (Dittmer and Misteli 2011). We therefore resist calling *C. elegans* either an A- or B-type lamin. As the reviewer states, many call LMN-1 a B-type lamin because it is constitutively farnesylated and is expressed in undifferentiated tissues. We attempt to explain this briefly on lines 79-83 of the revised manuscript.

5. In Figure 5, it is not clear why only the one mutant was analyzed, when others had a nuclear migration defect? Specifically, you attribute the migration phenotype to a possible defect in LMN-1::LINC interaction, but without checking several lmn-1 mutants that do or do not display a migration defect this correlation is premature.

We focused on the LMN-1(R204W) mutant because it had an almost completely penetrant nuclear migration defect and no other significant defects. This led to our hypothesis that R204W specifically disrupts the interaction between LMN-1 and the SUN protein UNC-84, while not disrupting the other functions of LMN-1. The other alleles with significant nuclear migration defects (N53S and Y59C) are partially penetrant and have other defects consistent with the hypothesis that they are general partial loss of function alleles that disrupt multiple LMN-1 functions.

Moreover, there are technical difficulties doing this experiment in N53S or Y59C strains, because they are homozygous inviable. We measured the levels of nuclear envelope-localized, immunofluorescently-labeled UNC-83 in the R204W comma stage embryos in Figure 5A-B. This was not possible in N53S or Y59C mutants because the balancer GFP marker is not expressed in comma stage embryos. Thus, we have no way to identify a comma stage embryo from a N53S/balancer mother as homozygous mutant or heterozygous. The UNC-84::GFP experiment is also problematic. Our UNC-84::GFP is an insertion at the *unc-84* genomic locus, and is very faint. We really cannot measure its intensity well; it is more of an all or nothing assay, and it is not observable at all in embryos without counterstaining with anti-GFP antibodies. We did cross the UNC-84::GFP into N53S or Y59C backgrounds and the GFP was below detectable in most homozygous cells. Yet, there must be some UNC-84 present in the nuclear envelope, as about 2/3 of nuclei migrate normally, and we did see nuclear envelope-localized UNC-84::GFP in some small, non-hypodermal, nuclei in adults. Given the complex nuances of these data, we chose to focus in the manuscript on the R204W mutant, as that is the most relevant for our hypothesis that it represents a separation of function allele.

6. The comments regarding statistical analyses, as noted by Reviewer #1, should be addressed. It might help is you explained why different tests were applies in the Figures with multiple comparisons to the wild type sample.

See below.

7. A few statements should either be elaborated on or toned down, as suggested by both reviewers.

See below.

8. Why does Table 1 not include the micronuclei phenotype? That would “elevate the status” K284Q allele.

We went back and forth about including nuclear morphology in the scoring system. However, we have not determined if the nuclear morphology defect is a result of defects in mitosis of hypodermal cells. If the nuclear morphology defects were a result of mitotic defects, we would likely have already scored this in the embryonic lethality category. Second, we do not understand why the N53S and Y59C heterozygotes have a more severe defect than the homozygous animals. Perhaps this is consistent with the disease states, as the *LMNA* mutations are dominant. But it could also mean that this is a phenotype related to embryonic lethality, and the heterozygotes have more nuclear morphology defects than the homozygotes because the more severe homozygotes die during embryogenesis? Finally, we are hesitant to “elevate the status” of the K284Q allele. While it has a significant nuclear morphology defect, it is very weak, the weakest we observed. We recognize that this explanation is vague and diffuse, which is why we did not include it in the discussion.

**Reviewer #1:**

The manuscript by Gregory and colleagues describes the phenotypes of a series of C. elegans mutants designed to mimic missense mutations in the human LMNA gene. LMNA encodes lamin A and lamin C proteins that are components of the nuclear lamina in all differentiated cell types. A remarkably high number of LMNA mutations are causatively linked to a diverse set on human diseases known as laminopathies. Despite intensive research efforts, it is largely unknown how the individual changes in lamin A (and lamin C) lead to clinical manifestations in specific tissues. In this manuscript, the authors focused on 5 autosomal dominant mutations that cause both cardiac and skeletal muscle defects, 3 mutations linked to cardiac muscle defects only and 4 mutations that the authors categorized as variants of unknown clinical significance (VUS). All 12 mutations are missense mutations that affect a single amino acid residue conserved between the human lamin A and C. elegans LMN-1 proteins: CRISPR/Cas9 genome engineering was used to introduce the relevant mutations into the endogenous lmn-1 gene.

The 5 mutations in the first category caused a severe reduction in viability and fertility, whereas the mutations in the two other categories had no or little effect on these parameters. To evaluate muscle activity, worms were placed in liquid medium and body bends per second were measured. This revealed reduced activity for 3 of the mutants in the first category and for 1 of the VUS. Nuclear migration defects were observed for 3 mutants; delocalization of LINC (Linker of the Nucleoskeleton and Cytoskeleton) complex members was evaluated and observed for 1 of these mutants. Finally, nuclear morphology was found to be abnormal in several mutants. Combining the data on viability, swimming and nuclear migration a score was assigned to each mutant. Although the mutants show variability within each parameter, the combined score separated the 5 severe mutations (causing cardiac and skeletal muscle defects in humans) from the remaining mutations.

The manuscript is technically very sound, well written and will be of interest to many colleagues studying nuclear organization, laminopathies and/or muscle function. The modification of the endogenous lmn-1 represents an important improvement compared to most previous studies. However, I suggest the authors to consider the following points prior to publication:

- Some claims seem unjustified. For instance, “we uncovered molecular mechanisms for how lamins interact with other nuclear envelope proteins to carry out their cellular functions” and “The modeled missense mutations also uncovered new mechanistic insights into the normal roles of lamin in development.” It is not clear to me which are the novel molecular mechanisms.

We agree with the reviewer that some conclusions about the R204W variant were overstated. We have made the following changes to tone down our conclusions: The last sentence of the Introduction on lines 107-108 now reads “revealing a separation of function allele that could provide insights into lamin function during nuclear migration.” We deleted the sentence “Furthermore, we uncovered molecular mechanisms for how lamins interact with other nuclear envelope proteins to carry out their cellular functions.” from the Author Summary. We also deleted the sentence “The modeled missense mutations also uncovered new mechanistic insights into the normal roles of lamin in development.” from the first paragraph of the Discussion. We also added a qualifier “that is predicted to” to the penultimate sentence on line 380 in the Discussion.

- The manuscript should describe the differences between A and B type lamins. Since the first publication describing its role in C. elegans, LMN-1 has been termed a B-type lamin. According to Uniprot, the identity between LMN-1 (Q21443) and LMNB1 (P20700) is 31.3% whereas the identity between LMN-1 (Q21443) and LMNA (P02545) is 29.4%. Like B type lamins in humans (and other vertebrates), LMN-1 is presumably permanently farnesylated, whereas lamin A proteins is posttranslationally modified to remove the farnesylated C terminus. This does not necessarily influence the conclusions of the manuscript but the authors should explain to the readers that the expression and processing of lamins are more complex in humans as compared to C. elegans.

See response to Editor query #4 above.

- The information in lines 101-106 should be updated to mention that Penfield and colleagues expressed and evaluated an un-tagged, single-copy LMN-1(N209K) mutant in a lmn-1(0) background (doi: 10.1091/mbc.E17-06-0374).

We have added a discussion of the Penfield et al N209K model to lines 95-97 of the Introduction. This is the closest model to expressing mutant lamins at physiological levels and we apologize for not including it previously. We now write starting at line 91, “Many published models express missense variant LMN-1 proteins from multi-copy extrachromosomal arrays in the presence of the intact endogenous *lmn-1* locus [30–34]. Consequently, the relative levels of mutant and wild-type LMN-1 are difficult to accurately measure and may vary from tissue to tissue or cell to cell. In addition, most models fuse LMN-1 to fluorescent proteins or tags that have been demonstrated to disrupt lamin function *in vivo* [30,33,35]. One model utilized an untagged, single-copy of LMN-1(N209K), which is likely to better model the human disease. However, analyses of this strain focused on the early embryo [36].”

- One on the main arguments by the authors is that C. elegans is an attractive model to evaluate VUS. Of the 4 putative VUS tested in the manuscript, R331Q showed a swimming defect. However, based on references 60 and 66, is R331Q indeed a VUS? Ref 66 concluded that “Clinical, morphological, functional, haplotype, and segregation data all indicate that LMNA p.(Arg331Gln) is a pathogenic founder mutation” and in the ClinVar databased R331Q is listed as “ Pathogenic(8); Likely pathogenic(1); Uncertain significance(1)”. I suggest the authors to consider is R331Q classifies as a VUS. I think the argument that C. elegans is an attractive model to evaluate VUS compared to for instance vertebrate models in terms of cost, speed, bioethics, etc. is still equally valid.

Assigning a *LMNA* variant to be disease causing or a VUS is not clear cut. We thank the reviewer for pointing out that reference 68 (was originally 66) previously identified R331Q as disease causing. We missed this. ClinVar is not so clear. We looked this week, and as of the last annotation (May 15, 2023), ClinVar lists R331Q as “conflicting interpretations of pathohgenicity.”

We therefore treated R331Q as a VUS for the purposes of this study. We now explicitly state that Ref 68 already predicted that R331Q is disease causing in three places. (1) On lines 144-146 in the results, we now include the following sentence: *LMNA* pR331Q is associated with DCM-SD [68], but remains annotated as in the ClinVar database [62] as a variant with conflicting interpretations of pathogenicity. We therefore categorized pR331Q as a VUS for the purposes of this study.” (2) On line 220 of the results, we state: “K331 has been linked to DCM-CD (Table1 and [68]), validating our approach for predicting VUS pathogenicity.” (3) On lines 332-333 of the Discussion, we have the following: “in agreement with previous predictions [68], our scoring system predicts that one *LMNA* VUS (p.R331Q) is pathogenic”

- The p values in Figure 2A-B should be corrected for multiple comparisons as in Fig3B. Similarly, which test was used in Figure 4C? Was correction for multiple comparisons applied?

We have addressed these concerns and the statistical tests we used are now better described in the revised figure legends.

- The heading in line 192 should be corrected: only 4 of 12 had impaired swimming behaviour.

Based on this suggestion, we changed the heading, now on line 185, to “Some *lmn-1* missense mutants had impaired swimming behavior”

- In several occasions the genotype-phenotype correlation is not straight forward. For instance, Y59C and L535P correspond to dominant mutations in humans. In the swimming assay, heterozygous and homozygous Y59C mutants behave similar whereas homozygous L535P mutants are more affected than heterozygous L535P mutants. In contrast, in terms of nuclear morphology, the Y59C mutation affects heterozygous animals more than homozygous individuals whereas heterozygous and homozygous L535P mutants behave similar. The behaviour of Y59C in latter assay is discussed superficially in lines 348-351. I think the authors should discuss this in greater detail. Could the balancer chromosome affect the nuclear morphology assay?

See response to Editor query #4 above.

- Also regarding the evaluation of nuclear morphology: The reporter should be described in more detail. Which NLS was used, how many copies, etc? Without a nuclear envelope marker, can the authors confidently distinguish between micronuclei (i.e. separated from main nucleus) or nuclear blebs when the GFP signal is close to the main nucleus? The potential presence of chromatin bridges without using a chromatin marker is not convincing.

See response to Editor query #3 above.

- The observations reported in Figure 5 are interesting, but why was only a single mutant analysed and not all three mutants with nuclear migration defects?

While we would have loved to do this analysis in the other mutations, we could not, due to technical reasons. Most importantly, we cannot do the immunofluorescence to UNC-83 in the balanced mutations because we cannot identify embryos are heterozygous at the stage we analyzed the images. For further information, see response to Editor query #5 above.

- By IF, there is less UNC-83 in the nuclear envelope of cells of R204W embryos whereas UNC-84::GFP seems completely absent in the hypodermis of R204W adults. Is the difference in behaviour of UNC-83 vs UNC-84 due to the method (IF vs live), the stage (embryos vs adult) or because they are different proteins?

We think this difference is because the UNC-83 is antibody staining and the UNC-84 is live GFP. The UNC-84::GFP is very faint. For more of an explanation, see response to Editor query #5 above.

- Finally, there seems to be less LMN-1(R204W) at the nuclear envelope. This would be relevant to document by quantification.

Thank you for this suggestion. We have now more carefully measured the IF staining of LMN-1 in wild type and the *lmn-1(R204W)* mutant. We did not see a significant difference. We now include this data in the new Figure 5B and discuss the new data in the results on lines 258-260. “To determine whether the *lmn-1(R204W)* variant disrupts the interaction between the LINC complex and LMN-1, we examined the nuclear envelope localization of UNC-83, UNC-84, and LMN-1 (Fig 5). We did not detect a significant change in LMN-1 localization (Fig 5B).”

- The legend to Figure 6B mentions p values but these are not represented in the Figure.

We found that adding all the *** to the figure made it complicated. We now state the following in the figure legend on lines 303-305: “All the genotypes are statistically significantly different from wild type, except for the hT2/+ control and the five marked n.s. for not significant.”

**Reviewer #2:** 
In their manuscript, Gregory et al successfully employ CRISPR/Cas9 genome editing techniques to produce a variety of nematode lines, each containing different human LMNA mutations. These mutations span a wide range, from those known to cause muscle disorders to those whose clinical implications remain unclarified. Through the use of a comprehensive selection of established testing methods, applied at both the cellular and organismal levels, the authors convincingly demonstrate that these mutations give rise to diverse phenotypes. Importantly, these phenotypic expressions generally align with the known severity of the corresponding human diseases. Remarkably, certain mutations previously categorized as clinically ambiguous also yield significant phenotypes. This study thus provides a straightforward approach to potentially ascertain the clinical impact of newly discovered variants. Given its implications, it is my belief that this work is well-suited for publication in PLoS Genetics.

Major comments:

- The major area of concern in this study relates to the controls used to account for genetic background. It remains unclear whether the animals that were engineered underwent any out-crossing to exclude the potential influence of nonspecific editing.

See response to Editor query #1 above.

- Additionally, in all conducted tests, balanced mutants were compared directly with wild-type animals. However, these balanced mutants not only carry a single copy of the mutation but also contain the balancer construct. The potential impact of this balancer on the results should be addressed and verified. Without these clarifications, there might be an underlying risk of attributing observed phenotypes to the specific mutations, while they might be influenced by other genetic factors.

See response to Editor query #2 above.

Minor comments:
1. While the schematic model in Figure 1C provides valuable insights, the manuscript would be significantly enhanced by the inclusion of a more detailed structural model that maps these mutations to LMN-1. Tools such as AlphaFold, among others, can be utilized to generate these intricate and realistic structural models with relative ease.

We have experience with this for other projects and tried to generate Alphafold (or Collabfold) models for LMN-1 but failed. All the models had extensive unstructured domains making them unhelpful for the reader. We felt that including any of them would be confusing to the reader. Here is an example of us trying to model a dimer of LMN-1, it just wants to fold back on itself and is not helpful to the reader. We do not want to include this in the manuscript.

2. Previous studies have reported the occurrence of micronuclei in cell lines expressing specific LMNA mutations. Consequently, it is crucial for the manuscript's discussion section to address this observation. It should also evaluate and elucidate on the presence or absence of evidence pertaining to micronuclei formation in the chosen mutations for this study. Furthermore, an analysis highlighting the pros and cons of using nematodes over cell lines should be provided.

As suggested, we have added the following to lines 352-355 of the Discussion: “The presence of micronuclei, chromatin bridges, and nuclear blebs has been reported in mammalian tissue culture models of laminopathies [77,78]. We saw similar nuclear morphology defects, again suggesting that *C. elegans* is a good model for studying laminopathies.” Also, see our comments in response to Editor query #2 above. As to a discussion of the pros and cons of nematodes vs. cell lines, we feel this was done in the paragraphs from lines 67-97 in the Introduction.

3. The scale bar at 3A appears to be wrong, as it suggests animals are <0.3mm long.

Thank you for catching that. We have fixed the scalebar in 3A.

4. Figure 3C needs error/confidence bars

The data in Fig 3C represent a percentage of all the animals we observed; therefore, it does not have an error bar. It's the same data as in 3B, but represented differently. 3B does a better job of showing the variance in the data.

5. In the water motility assay, Y59C, R64P and N53S had similar averages in het and homozygous state. This should be explicitly mentioned and discussed, similarly to the way it was discussed for the micronuclei.

We added the following sentence about this in the Discussion on lines 344-346: “For example, in our motility assay, Y59C, R64P, and N53S heterozygous and homozygous strains had similar average swimming rates.”

6. The statement, "However, animals that survive due to a presumably low level of RNAi," makes an assertion without robust mechanistic evidence to support it. A more measured speculation could be to suggest that survival is potentially due to increased residual protein levels.

We have changed the sentence on lines 223-224 to read: “However, animals that survive, potentially due to higher residual protein levels…”

7. How were the strains validated? If by Sanger sequencing, it should be mentioned in the methods section.

Yes, all lines were validated by Sanger sequencing. We have added this to line 406 of the Methods: “and all variants were validated by Sanger sequencing.”
